# *Mycobacterium bovis* PknG R242P Mutation Results in Structural Changes with Enhanced Virulence in the Mouse Model of Infection

**DOI:** 10.3390/microorganisms10040673

**Published:** 2022-03-22

**Authors:** Fábio Muniz de Oliveira, Victor Oliveira Procopio, Gabriela de Lima Menezes, Roosevelt Alves da Silva, André Kipnis, Ana Paula Junqueira-Kipnis

**Affiliations:** 1 Tropical Pathology and Public Health Institute, Federal University of Goiás, Goiania 74605-050, Brazil; fabiomuniziptsp@gmail.com (F.M.d.O.); victor.goler16@gmail.com (V.O.P.); akipnis@ufg.br (A.K.); 2 Department of Biomedical Sciences, Faculdade Estácio de Sá de Goiás—FESGO, Goiania 74063-010, Brazil; 3 Biosystem Collaborative Nucleus, Federal University of Jataí, Jatai 75804-020, Brazil; gabrieladelima_@hotmail.com (G.d.L.M.); rooseveltfisicaufg@gmail.com (R.A.d.S.)

**Keywords:** molecular dynamics, pathogenicity, macrophages, virulence, neutrophils, SNP, protein kinase

## Abstract

*Mycobacterium bovis* is the causative agent of tuberculosis in domestic and wild animal species and sometimes in humans, presenting variable degrees of pathogenicity. It is known that PknG is involved in the first steps of *Mycobacterium tuberculosis* macrophage infection and immune evasion. We questioned whether *M. bovis* *pknG* genes were conserved among mycobacteria and if natural genetic modifications would affect its virulence. We discovered a single mutation at a catalytic domain (R242P) of one *M. bovis* isolate and established the relation between the presence of R242P mutation and enhanced *M. bovis* virulence. Here, we demonstrated that R242P mutation alters the PknG protein conformation to a more open ATP binding site cleft. It was observed that *M. bovis* with PknG mutation resulted in increased growth under stress conditions. In addition, infected macrophages by *M. bovis* (R242P) presented a higher bacterial load compared with *M. bovis* without the *pknG* mutation. Furthermore, using the mouse model of infection, animals infected with *M. bovis* (R242P) had a massive innate immune response migration to the lung that culminated with pneumonia, necrosis, and higher mortality. The PknG protein single point mutation in its catalytic domain did not reduce the bacterial fitness but rather increased its virulence.

## 1. Introduction

*Mycobacterium tuberculosis* complex bacteria (MTBC) species have 11 genes coding for serine/threonine protein kinases [1] of which protein kinase G (PknG) is in the cytosol and exist as a dimer [2]. The PknG protein of the *Mycobacterium* genus has four domains with different length descriptions: unstructured N-terminal domain (aa 1–98), a rubredoxin domain (Rbx) (aa 99–147) that modulates the enzyme activity according to redox potential and is related to latency responses, a kinase catalytic region (aa 138–405), and a tetratricopeptide repeats domain (TPR) (aa 406–750) responsible for the dimerization of the protein. There are five well-defined regions within the catalytic domain: p-loop (158 to 164), helix c (186 to 205), catalytic loop (275 to 280), DGF motif (292 to 293), and the activation loop (296 to 302) [3,4].

Recently, Zulauf et al. [5] showed that PknG is secreted by Mtb during macrophage infection by a SecA2 dependent protein export system. PknG is involved in mycobacteria metabolic regulation by controlling the state of GarA phosphorylation and plays a crucial role in the survival of mycobacteria in macrophage hosts, blocking the intracellular degradation of mycobacteria in lysosomes, favoring their survival during hypoxia [6,7]. In addition to several mycobacterial proteins involved in the inhibition of the phago-lysosomal bacterial clearance [8,9], *M. tuberculosis* and *M. bovis* PknG was shown to interact with host Rab7 protein and this interaction, together with the activity of PknG, avoid phago-lysosomal fusion and maturation resulting in enhanced infection outcome [10]. Nakedi et al. [11] also identified several new targets of *M. bovis* BCG PknG, related to the mycobacterial metabolism such as TCA cycle enzymes and ATP binding proteins.

*M. bovis* is the causative agent of bovine tuberculosis, a disease with great impact in livestock worldwide, implying large economic losses [12]. In addition, this bacillus is also pathogenic for humans as well as for several domestic and wild species [13]. *M. bovis* belongs to the MTBC whose members have more than 99% identity between their genomes [13]. Despite their similarity, they manifest different pathogenic phenotypes in addition to different host specificities, due to extensive long-term co-evolution of mycobacterial species with various species of animals and humans [14].

Although *M. bovis* clinical isolates seem to have varied pathogenicity depending on the murine model of infection [15,16,17], other studies have shown that *M. bovis* isolated from different geographic regions showed significant differences in their virulence [18,19], suggesting that factors inherent to the mycobacterial strains play an important role in pathogenicity. As PknG was shown to be important for mycobacterial survival within macrophages stressful conditions and that it is involved in the process of phago-lysosomal fusion evasion, we posited that PknG variability may influence in mycobacterial infection outcome.

## 2. Materials and Methods

### 2.1. Alignment of PknG Protein Sequences from M. bovis Genome Sequences

The following amino acid sequences were obtained from the National Center for Biotechnology (https://www.ncbi.nlm.nih.gov/protein (accessed on 11 August 2021)): *Mycobacterium bovis* strain 1 (Mbt) (QEF46054.1); *Mycobacterium bovis* strain SP38 (ANG90777.1); *Mycobacterium bovis* strain 30 (AMC53676.1); *Mycobacterium bovis* strain 1595 (AKQ99979.1); *Mycobacterium bovis* strain 2002/0476 (AVK91932.1); *Mycobacterium bovis* BCG strain Mexico (AET17708.1); *Mycobacterium bovis* BCG strain Moreau RDJ (CCC63010.1); *Mycobacterium bovis* BCG strain BCG-1 (Russia) (ALV09656.1); *Mycobacterium bovis* BCG strain Tokyo 172 (AMO12216.1); *Mycobacterium bovis* strain Danish 1331 (QCU59836.1); *Mycobacterium tuberculosis* strain Beijing (AUS63684.1); and *Mycobacterium tuberculosis* H37Rv (CCP43141.1). Sequence alignment was performed with Clustal X v2.1 software (Dublin, Ireland), and the Clustal alignment was viewed with Jalview v2.0 software (Dundee Scotland, UK).

### 2.2. Mycobacterium bovis Isolation

Two *M. bovis* isolates were isolated from animals in the State of Goiás, Brazil. The first *M. bovis* (Mbb) isolate was obtained from an asymptomatic dairy cow (*Bos taurus*) with high milk productivity but with positive tuberculin skin test. After the animal was euthanized, granulomatous lesions suggestive of tuberculosis were collected from the lungs. At the Molecular Bacteriology Laboratory at Federal University of Goiás, the organs were processed and plated on Stonebrink media for mycobacteria isolation. Similarly, the other isolate (Mbt) was obtained from the lungs of a tapir (*Tapirus terrestris*) that died with severe classic symptoms of tuberculosis. The isolates were deposited in the Brazilian Culture Collection of the Institute of Tropical Pathology and Public Health, WDCM no. 1223.

### 2.3. Mycobacterial Isolate Preparation

Cultures of Mtb H37Rv, Mbb, and Mbt in 7H9 medium containing 0.05% Tween 80 and 10% oleic acid–albumin–dextrose (OAD) were grown until they reached exponential phase. Aliquots were made of these cultures and frozen in 10% glycerol at −80 °C. The concentration of Mycobacteria stocks was determined 30 days after being frozen by plating serial dilutions of each isolate on 7H11 supplemented with 10% OAD, by counting the colony-forming units (CFUs).

### 2.4. Mycobacterium bovis Mbt Isolate Genome Sequencing and Annotation

The full genome sequencing of the *M. bovis* Mbt isolate was performed using the Illumina HiSeq2500 strategy and 2 × 100 paired-end coverage reads. The quality of reads was evaluated using the software FastQC v. 0.11.5 (Babraham, Cambridgeshire, United Kingdom). Reads with low quality and adaptors were filtered by Trimmomatic v. 0.33 software (Los Angeles, CA, USA). The filtered reads were then assembled with the program Velvet [20]. The generated contigs were mapped by the ABACAS program (Algorithm-Based Automatic Contiguation of Assembled Sequences) and remaining gaps were closed using the software IMAGE (Iterative Mapping and Assembly is Gap Elimination) [21] and using the genome *M. bovis* AF2122/97 (NC_002945.4) as reference. The quality of the genome obtained was evaluated by QUAST (Quality Assessment Tool for Genome Assemblies) [22]. The generated genome was submitted to NCBI BLASTn program (https://blast.ncbi.nlm.nih.gov/Blast.cgi (accessed on 8 January 2020)), the annotation of the *M. bovis* Mbt genome was done by Prokka v. 1.12 software [23] and the sequence was given the ID of CP040832.1.

### 2.5. Confirmation of pknG Gene Mutation by Partial Gene Sequencing

The mutation in the *pknG* gene of Mbt and Mbb *M. bovis* isolates deposited in the Brazilian Culture Collection of the Institute of Tropical Pathology and Public Health, WDCM no. 1223, were evaluated for the presence of SNPs by partial gene sequencing. For that, primers (Fwd_pknG_SNP 5′ GTCGTTGTAGACCAAGCCGA 3′ and Rev_pknG_SNP 5′ CAACTTTGTCGAGCACACCG 3′) were designed using as reference the genome of *M. bovis* AF2122/97 for PCR and sequencing. The PCR was performed in a final volume of 50 μL containing 20 ng of DNA, 0.2 µM dNTP, 1U Taq DNA polymerase (Promega, Madison, WI, USA) and 1 µM of each primer using a thermal cycler (Biocycler MJ96G; Applied Biosystems, Foster City, CA, USA) under the following conditions: denaturation at 94 °C for 5 min; 35 cycles of denaturation (92 °C, 1 min), 45 s at the melting temperature of each pair of primer, and extension (72 °C, 1 min); a final extension step at 72 °C for 10 min. PCR products were separated by electrophoresis in 1.5% agarose gel containing 0.5 μg/mL of ethidium bromide and visualized using Gel Doc XR System (Bio-Rad Laboratories, Hercules, CA, USA). For sequencing purpose, the PCR products were precipitated by the addition of NaCl to a final concentration of 0.3 M, and 2.5 volumes of ice-cold ethanol, and following incubation at −20 °C overnight. Precipitated PCR product was recovered after centrifugation at 12,000× *g* for 20 min and the sediment was washed with 70% ice-cold ethanol. The sediment containing the purified product was resuspended in 25 µL of MiliQ water and the quality was analyzed by agarose gel electrophoresis. All the PCR products were sent for sequencing with each individual primer at ACTGene (https://actgene.com.br (accessed on 5 February 2020)).

### 2.6. Molecular Dynamics Simulations

In order to evaluate the structure implications of R242P mutation in PknG protein, molecular dynamics (MD) simulations were performed. The amino acids sequences from PknG proteins of *M. bovis* Mbt (strain QEF46054.1) and Mbb (strain QCU59836.1) were submitted to I-TASSER server [24] for structure predictions. The output models had their quality evaluated by the MolProbity server [25].

After model quality validation, protonation state of histidine amino acid for pH 7.4 was predicted by the PropKa [26] server and the initial steps for molecular dynamics were executed using the GROMACS 5.1.2 software [27] and AMBER99SB-ILDN force field. For both proteins, with and without mutation, the same molecular dynamics parameters were used. Thus, for each protein, the initial structure was inserted into a cubic box with a minimum distance of 10 angstroms (Å) between any box edge and protein atom. This box was solvated with TIP3P water, and the system was neutralized with sodium ion. The SETTLE algorithm [28] was used to maintain the internal rigid structure of solvent (water) molecules while solute covalent bonds involving hydrogen atoms were constrained by the LINCS algorithm [29]. The system temperature was adjusted to 300 K (26.85 °C) and pressure to 1 atm, both parameters were regulated by Berendsen et al. [30] and Parrinello-Rahman et al. [31] algorithms, respectively. For non-bonded interactions, a cutoff of 1.0 nm was defined and the particle mesh Ewald summation method [32] was applied for long-range electrostatic interactions. Leap-frog algorithm [33] was used to integrate MD motion equations using 2fs as a time step. Initially, the system was submitted to two energy minimization steps. The first one was performed with 500 steps of the steepest descent algorithm and protein position restriction. The second minimization step was done using the same algorithm but with 10,000 steps with flexible water.

After minimization steps, the system was submitted to equilibration steps consisting of two 100 picoseconds (ps) phases: an NVT ensemble and an NPT ensemble with protein position restriction for thermodynamic variables equilibration. The third and last equilibration phase was conducted as an NPT ensemble of 1 nanosecond (ns) without protein restriction. Finally, the production run was carried out as an NPT ensemble at 300 K within the total time of 200 ns. For each protein, those procedures were performed in duplicates, resulting in two molecular dynamics simulations for protein with and without mutation. Trajectory analyses were performed using GROMACS 5.1.2 tools.

### 2.7. Evaluation of M. bovis Isolates Growth under In Vitro Stress Conditions

For in vitro growth comparisons, Mtb and *M. bovis* isolates were cultured to log phase (15 days) in 7H9 medium supplemented with OAD. Cultures were washed with PBS containing 0.05% Tween 80 and OD_600_ was adjusted to 0.04 in 7H9 medium under different conditions. For acid growth evaluation, the 7H9 medium had the pH adjusted to 4.5 with 4 M hydrochloric acid. For oxidative stress, the medium was supplemented with 5 mM hydrogen peroxide. For evaluation of antibiotic resistance, 5 μg/mL of isoniazid was added to the culture medium. The growth of mycobacteria under different conditions was measured at OD_600_ during 6 days of incubation at 37 °C and 140 rpm shaking.

### 2.8. Infection of Bone Marrow-Derived Macrophages (BMDM)

The bone marrow of C57BL/6 mice was collected and subjected to differentiation as previously described [34]. BMDM (1 × 10^6^ cells/mL) were infected with mycobacteria at a MOI of 1:1 in a 24-well plate. Three hours after the infection, the extracellular bacteria were removed by washing the wells twice with RPMI supplemented with 10% fetal bovine serum and then the cells were cultivated with complete RPMI or with RPMI medium containing 500 μM DFO to remove free iron. Forty-eight hours later, the BMDM were washed with medium, and cells were lysed with nuclease-free water (Ambion). The lysate was transferred to 1.5 mL nuclease-free tubes, centrifuged at 16,000× *g* for 10 min at 4 °C, and the pellet was processed for RNA extraction and genes expression evaluation by RT-PCR [34]. Ct values were tabulated on an Excel 2011 spreadsheet, and the relative expression of the *pknG* gene was determined by the delta delta Ct (2^−ΔΔCt^) method using the expression of the *sigA* gene as normalizer. Additionally, infected BMDM for 48 h, as described above, had their supernatant and cell lysate evaluated for the presence of extravasated and intracellular mycobacteria, respectively, by plating serial dilutions on 7H11 supplemented with OAD and pyruvate for evaluation of the CFU.

### 2.9. Animals

Eight weeks old C57BL/6 female mice were obtained from the animal facility of the Tropical Pathology and Public Health Institute (Instituto de Patologia Tropical e Saúde Pública—IPTSP). Mice were kept in micro isolators adapted to racks with a HEPA filter and subjected to cycles of 12 h of light/darkness at 24 °C temperature and 50% humidity. The animal experiments carried out in this study were approved by the Ethics Committee on the Use of Animals at the Federal University of Goiás (Protocol number: 060/17).

The infection experiment consisted of three groups of 20 animals each. Each group was infected by one of the mycobacteria strains. One infected mouse from each group was used for the determination of the infection load, and for each time point during infection, 3 animals per group were euthanized at a time. The remaining 10 mice from each group were used for survival rate determination. The experiments were repeated three times.

### 2.10. Animal Infection, Survival, and Bacterial Load Determination

Infection was performed by thawing a vial of each strain, diluting them in PBS to reach the concentration of 10^7^ CFU/mL, and 100 µL of each inoculum was injected intravenously into the tail vein of mice. To determine the initial bacterial load, a mouse from each group was euthanized one day after infection, and lungs were homogenized and plated on 7H11 agar supplemented with OAD and pyruvate. About 21 days after plating, the infection dose was determined.

After the periods of 5, 15, and 21 days post-infection (p.i.), the animals were euthanized, and the right lung lobes, liver, and spleen were collected, homogenized, serially diluted, and plated onto 7H11 agar supplemented with OAD and pyruvate to verify the bacterial loads. Ten animals per group were infected as above and followed for 30 days to check for mortality. The animals were observed daily for any sign of severe symptoms, and if so, were humanely euthanized.

### 2.11. Evaluation of Pulmonary Pathology

The accessory (right caudal) pulmonary lobe was collected from each mouse in periods of 5, 15, and 21 days p.i., perfused, and fixed with 10% paraformaldehyde. Five-micrometer-thick histological sections from each organ were stained with hematoxylin and eosin (H&E) and evaluated in a light microscope (Axioscope A1; Carl Zeiss, Jena, Germany). The lesion scores were determined based on the areas with injuries in relation to the overall visual field organ on 10× objective magnification with the help of the software AxioVision microscopy. The results were expressed as a percentage of areas with injury. Four to six different fields from each lung were randomly selected for each animal organ (*n* = 6) from each group and the lesions were measured.

### 2.12. Flow Cytometry Analysis

Evaluation of the cellular recruitment to the lungs of infected mice with the different strains was performed 5, 15, and 21 days p.i. Six animals from each group were euthanized by cervical dislocation and the left lung lobes were collected. After digestion of the lobes with DNAse and collagenase, the organ was passed through a 70 µm nylon sieve (BD Biosciences; San Jose, CA, USA) and treated with RBC Lysis buffer (0.15 mM and 10 mM NH_4_Cl KHCO_3_) to retrieve a single-cell suspension. The cells were washed with RPMI and the suspension concentration was adjusted to 10^6^ cells/mL. Cell staining was performed by distributing 200 µL of cell suspension in each well of a 96-well plate (Costar, Corning, NY, USA) and incubating with fluorescent-labeled antibodies. Cells were incubated for 30 min with CD11b antibodies conjugated with FITC (eBioscience M1/70), PE-conjugated anti-CD11c (eBioscience N418), APC-conjugated anti-F4/80 (Biolegend BM8), PercP-conjugated anti-Gr1 (Biolegend RB6-8C5), FITC-conjugated anti-NK1.1 (eBioscience PK136), and APC-conjugated anti-CD3 (Biolegend 145-2C11). Later, the cells were washed with PBS and fixed with paraformaldehyde using Perm Fix (BD Cytofix). A total of 50,000 events were acquired in the flow cytometer (BD Biosciences FACSVerseTM) and analyzed with FlowJo software 9.0. Positive events to F4/80^+^ were selected and the various cell populations were evaluated according to the presence of CD11b and CD11c markers. Alveolar macrophages were characterized as F4/80^+^CD11b^+^CD11c^−^, DC as F4/80^+^CD11b^+^CD11c^+^, neutrophils as F4/80^+^CD11b^high^CD11c^−^Gr1^+^ and monocytes/macrophages as F4/80^low^CD11b^low^CD11c^low^ according to Gonzalez-Juarrero et al. [35]. After the selection of the cell population according to size and granularity compatible with lymphocytes, cells marked with CD3 and NK 1.1 were evaluated. NKT cells were considered as CD3^+^NK1.1^+^ and NK cells as CD3^-^NK1.1^+^.

To evaluate the specific immune response generated by infection with the different mycobacterial strains, lung cells, obtained as described above, were stimulated with ConA (positive control) (1 µg/mL; Saint Luis, MI, USA), CMX (10 µg/mL of fusion protein composed of Ag85c, MPT51, and HspX proteins from *M. tuberculosis*; [36]), or media alone (negative control) and cultivated for 4 h in a 5% CO_2_ incubator maintained at 37 °C. Then, 3 µM of monensin (eBioscience; San Jose, CA, USA) was added to the culture, and the plates were incubated for 4 h. The plates were centrifuged, and the cells were incubated with anti-CD4 antibody conjugated with FITC (BD; clone IM 7) and an anti-CD8 PE-conjugated (BD; clone 53-6.7) for 30 min. Later, the cells were washed, fixed, and permeabilized using BD Cytofix kit/Cytoperm. After this step, the cells were incubated for 30 min with PercP-conjugated anti-IL-17 (eBioscience; clone eBio 17B1) and APC-conjugated anti-IFN-γ (eBioscience; clone eBio XMG1.2). Analysis of 50,000 events was carried out in a flow cytometer from the selection of lymphocytes according to size (FSC) and granularity (SSC). Cells stained with anti-CD4 or anti-CD8 were evaluated for positivity of IFN-γ or IL-17. The results were presented as mean and standard deviation for each group. The responsiveness of cell suspensions was compared with the positive and negative controls.

### 2.13. Statistical Analysis

The data obtained from the experiments were tabulated using Excel and Prism (Graphpad 4.0, San Diego, CA, USA) software packages. Growth curve comparisons were made using one unpaired t-test per time point using false discovery rates (FDR = 1%) and the two-stage step-up method of Benjamini, Krieger, and Yekutieli. Immune data were analyzed using one way ANOVA test to evaluate variance between groups followed by Mann–Whitney post test. To check if there were significant differences between the two groups, a nonparametric Wilcoxon–Mann–Whitney test was used. Survival data were analyzed by the logrank (Mantel–Cox) test. The differences were considered significant when *p* was < 0.05.

## 3. Results

### 3.1. M. bovis PknG Sequences Alignment and Tertiary Structures

**Figure 1:**
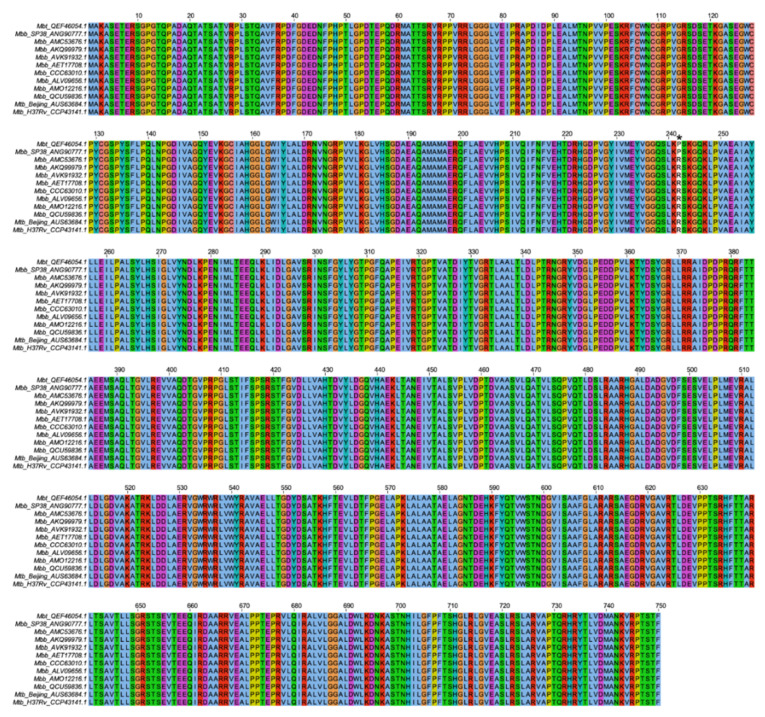
Presence of non-synonymous SNPs in PknG protein of genes within *M. bovis* isolates. The protein sequence of PknG proteins from *M. bovis* genomes deposited in Gene Bank as well as that from *M. tuberculosis* H37Rv and a representative Beijing strain were aligned with ClustalX. * The mutation R242P is shown with an asterisk.

Recent studies show that the PknG protein has a role in the virulence of pathogenic mycobacteria, and this is related to the escape of phagolysosomes in macrophage cells. Therefore, the conservation of this protein structure and function is important for pathogenic mycobacteria. To evaluate if the PknG protein is conserved in *M. bovis* isolates that had their complete genome deposited in GenBank, the protein sequences were aligned and as shown in Figure 1, the PknG protein is conserved in almost all analyzed genomes. Surprisingly, it was observed that two isolates presented a single amino acid substitution at position 242 from a charged arginine residue to a hydrophobic proline (R242P) residue. When *M. bovis* PknG sequence was compared with the *M. tuberculosis* PknG sequence, it was clear that the PknG was shown to be conservative, and the mutation was not shared by *M. tuberculosis* strains. Interestingly, this polymorphism occurred at a position comprising the catalytic site of PknG.

Thus, to verify if the R242P mutation would interfere with secondary and tertiary structures of the PknG protein, molecular dynamics (MD) simulations were performed. The model of the PknG protein from the Mbb isolate (QCU59836.1) obtained 82% and 92.1% of residues in favored and allowed regions, respectively. For the PknG protein from the Mbt isolate (QEF46054.1), these values were 82.8% and 92.4%, respectively (Figure S1). After initial steps of molecular dynamics (energy minimization, NVT and NPT ensembles), an increase in protein quality parameters was observed (Figure S1). These results suggest that the I-TASSER server predicated high-quality protein models and energy minimization increased the structure quality.

**Figure 2:**
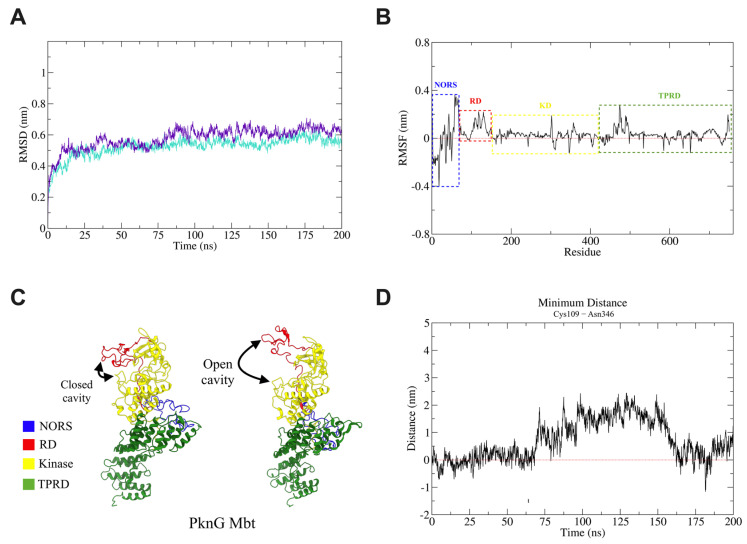
Molecular dynamics simulations analysis of PknG proteins. Molecular dynamics of the PknG protein from Mbb and Mbt isolates was performed in duplicates to evaluate protein structure and behavior in water. The main results suggest that PknG from Mbt has more opening conformation of substrate binding cleft than PknG from Mbb, which leads to higher kinase activity. (**A**) Average RMSD evolution of the PknG protein of Mbb (turquoise line) and Mbt (violet line) strains calculated from two independent 200 ns MD simulations for each strain. The individual RMSD values for each simulation can be seen in Figure S2B. (**B**) RMSF difference (Mbt − Mbb) of RMSF average calculated from two independent 200 ns MD simulations. Values above the red line (zero value) represent increased fluctuations in Mbt compared with Mbb isolates. Values below the red line represent decreased fluctuation in Mbt compared with Mbb isolates. Protein domains are delimited by a dashed rectangle according to the following colors: blue (NORS), red (RD), yellow (KD), and green (TPRD). (**C**) Structure of PknG showing open and closed cavity conformations. Protein domains are colored as previously described. (**D**) The distance of Cys109-Asn346 was calculated for each independent simulation (Figure S3B,C). The average for each isolate was obtained and the difference (Mbt − Mbb) resulted in the graph. Values above the red line represent a more open conformation of the PknG substrate binding site in the Mbt strain than in the Mbb strain. Values below the red line represent a less open conformation of the PknG substrate binding site in the Mbt strain than in the Mbb strain.

The root mean square deviation (RMSD) analysis from the MD trajectory of PknG proteins comparing the average of replicas between the proteins from both strains showed slightly higher RMSD values for the Mbt strain after ~78 ns (Figure 2A). These results suggest that the R242P mutation does not affect overall protein stability since on average a similar RMSD evolution behavior was observed between strains.

Root mean square fluctuation (RMSF) analysis demonstrated that replicas from the Mbb isolate had more RMSF match values than replicas from the Mbt strain (Figure S2A). It is important to note the difference in the RMSF average (aveRMSF_Mbt_ − aveRMSF_Mbb_) between the PknG protein from both strains (Figure 2B). The main difference appears at the regulatory secondary structure (NORS) region of the PknG protein (approximately residues 1–75). The same inference was made for the Kinase domain (KD), where only some isolated residues were seen with different fluctuations. In the tetratricopeptide repeat domain (TPR), a region (approximately residues ~450–500) with an increased fluctuation in the Mbt strain was observed. It is already known that this domain does not intrinsically affect kinase activity [37] and it is related to protein–protein interactions [2,38]. Further analysis should be conducted to determine how these fluctuations affect the interaction interface.

Unlike NORS and TPRD, the rubredoxin-like metal-binding motif (RD) regulates intrinsic kinase activity by restricting substrate-binding site access [37] (Figure 2C). A clear structural change was observed in the RD that may be related to increased kinase activity. The analysis of the difference in minimum distance between the residues Cys109 and Asn346 (Figure 2D and Figure S3) to assess how the substrate-binding cleft opens suggest that R242P mutation in PknG protein from the Mbt strain on average has a more open conformation of the ATP binding site cleft and this could result in an increased kinase activity (Figure S3A). This result correlates to previous studies where it was suggested that RD oxidation allowed a more open conformation of this same cavity and increased kinase activity [39].

**Figure 3:**
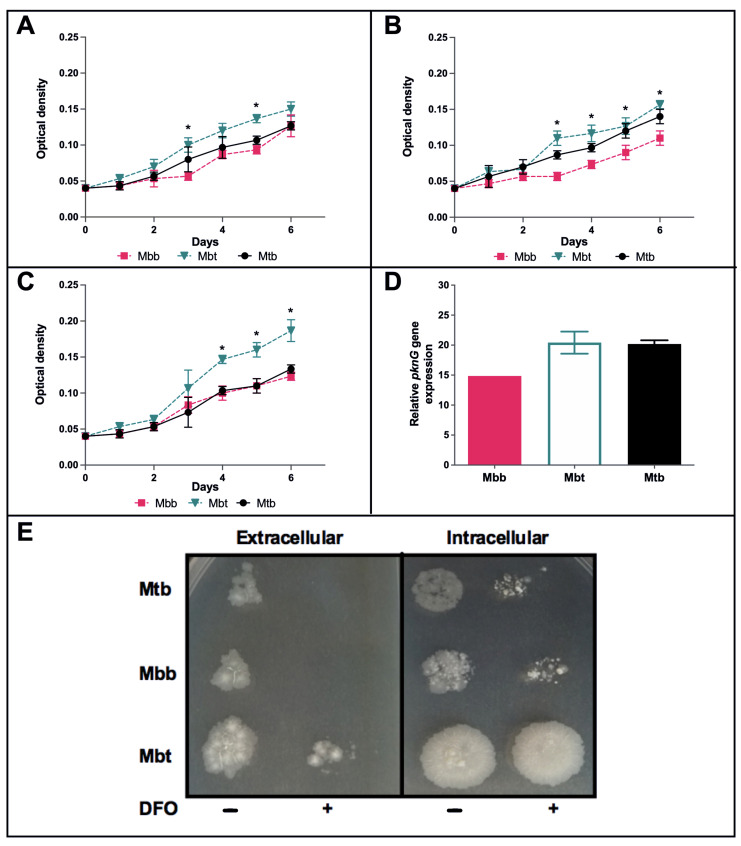
Mycobacterial growth under stressful conditions and *pknG* expression during macrophage infection. Bacteria were grown in 7H9 medium for 6 days under different conditions. (**A**) Cultures grown with 5 mM H_2_O_2_ (oxidative stress); (**B**) cultures grown under acid condition (pH 4.5); and (**C**) cultures grown in the presence of isoniazid (5 μg/mL). The results shown are the mean ± standard deviation of the different time points (*; significant difference, *p* < 0.05 between Mbt and Mbb). BMDM were infected at a MOI of 1:1 with Mtb, Mbb, or Mbt and three hours later extracellular mycobacteria were removed by replacing the supernatant media. (**D**) After 48 h of infection, macrophages were lysed, and intracellular mycobacteria were recovered. Expression of *pknG* gene was analyzed by quantitative RT-PCR with SybrGreen. The relative gene expression of *pknG* was determined by the delta delta Ct (2^−ΔΔCt^) method using the expression of the *SigA* gene as a normalizer. (**E**) After 48 h of infection, the supernatant (extracellular) and the attached macrophages were investigated for bacterial concentration by plating on agar 7H11 supplemented with OADC and pyruvate. Bacteria that extravasated from macrophages during infection were detected in the supernatant, while bacteria recovered from the lysed macrophages were considered as intracellular bacteria. Infected BMDM were supplemented with DFO (500 µM) or not to remove free iron when media was replenished 3 h after infection, and after 48 h of infection extravasated and intracellular mycobacteria were determined.

Thus, the R242P mutation could change the conformation of the ATP binding site cleft to be more open, suggesting that it could affect or improve the protein activity. Hence, we decided to evaluate whether or not such a mutation could affect the two *M. bovis* isolates from the Brazilian Culture Collection of the Institute of Tropical Pathology and Public Health (WDCM no. 1223) Mbt and Mbb, the former containing the R242P mutation. We investigated if such a mutation altered the ability of *M. bovis* to grow in stressful conditions such as the presence of hydrogen peroxide, acidic pH, or isoniazid [7]. Unexpectedly, the *M. bovis* Mbt isolate presenting such a mutation showed improved growth in all tested conditions (Figure 3A–C) compared with the *M. bovis* Mbb isolate without such mutation. Thus, it is suggested that this mutation in PknG might confer advantages for macrophage infection.

In this sense, we evaluated if BMDM infection with Mbt (R242P) would present greater macrophage lysis when compared with the other strains. As shown in Figure 3E, after 48 h of infection, cultures with Mbt-infected macrophages presented higher extracellular and intracellular bacillary loads than macrophages infected with the other isolates (Figure 3E, left lanes of each quadrant). These results could indicate that Mbt has greater resistance to the macrophage defense mechanisms, being able to grow more than the other mycobacteria. Interestingly, when macrophages were cultured with DFO, to further increase iron deprivation induced by macrophages, a reduction in the intracellular bacterial load was not observed in the Mbt group when compared with the cells not treated with DFO (Figure 3E; intracellular environment, right lanes of each quadrant). However, the extravasation of all tested mycobacteria was dependent on iron (Figure 3E; extracellular environment). Remarkably, despite iron absence, Mbt was still present in the extracellular environment while the other mycobacteria isolates were not. To evaluate if the stressful conditions during macrophage infection altered the PknG expression, the *pknG* mRNA levels were evaluated and showed no difference between the groups (Figure 3D). This result suggests that the phenotypic differences observed between the strains are not related to a compensatory increased level of *pknG* gene expression.

### 3.2. M. bovis Mbt Isolate (R242P) Have Increased Virulence in the Murine Model of Infection

**Figure 4:**
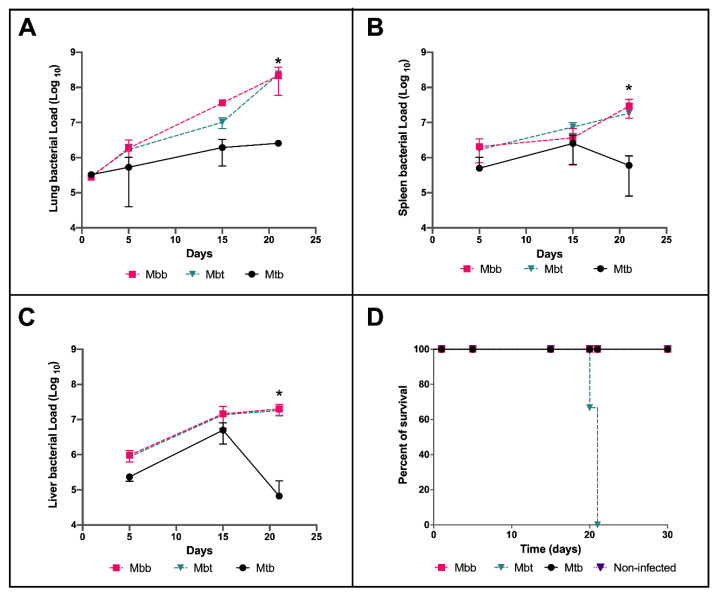
Evaluation of the mycobacterial load and survival of infected mice. C57BL/6 mice infected with 10^6^ CFU of *M. bovis* Mbb, Mbt, or Mtb strains were euthanized at 5, 15, and 21 days p.i. and the number of CFU in lungs (**A**), spleen (**B**), and liver (**C**) were determined after plating on 7H11 agar supplemented with pyruvate and OADC. In (**A**), the CFU recovered from the lungs at one day post infection is also shown. The lines indicate the kinetics of the mean ± standard deviation of the number of CFU from each strain obtained with three animals per group, showing the statistically significant difference with an asterisk (*). This experiment is representative of 3 independent experiments. (**D**) Mice survival curve for each infected group accompanied for 30 days.

Since infected macrophages resulted in a higher bacterial load of Mbt under stressful conditions, we postulated that this isolate would have higher virulence in the mouse model of infection. For that, C57BL/6 mice were infected with *M. bovis* isolates and the bacillary load was checked at 5, 15, and 21 days post infection (p.i.) and compared with the well-known infection of mice with *M. tuberculosis*. As shown in Figure 4, infection with the *M. bovis* isolates resulted in higher bacillary loads in all organs analyzed, mostly after 21 days of infection, when compared with Mtb (Figure 4A–C). In the lungs, the difference reached 2 log changes 21 days after of infection (Figure 4A). Nonetheless, there was no difference in the bacterial loads between the two *M. bovis* isolates. Mice survival analysis showed that although the bacillary load between *M. bovis* infected animals were similar in the various organs evaluated, mice infected with *M. bovis* Mbt isolate succumbed to death very early during infection—100% of the mice in this group died at 22 days p.i. (Figure 4D). These results demonstrated that despite the similar growth in the mice model of infection, the *M. bovis* isolates infection had different outcomes.

### 3.3. Innate Immune Responses Evoked by M. bovis Presenting Different PknG Proteins

To further understand the pathogenicity mechanisms that led to the distinctly observed virulence, the lungs of mice infected with the different isolates and at the different time points were evaluated by flow cytometry analysis. The cells of myeloid lineage, predefined as F4/80^+^, were selected based on the gating strategies and then classified based on expression of the additional markers as cellular macrophages (F4/80^+^CD11b^+^CD11c^−^), DC (F4/80^+^CD11b^+^CD11c^+^), neutrophils (F4/80^+^CD11b^high^CD11c^−^Gr1^+^), or monocytes/macrophages (F4/80^low^CD11b^low^CD11c^low^). The lymphoid lineages were identified as NKT cells (CD3^+^NK1.1^+^) or NK cells (CD3^−^NK1.1^+^).

**Figure 5:**
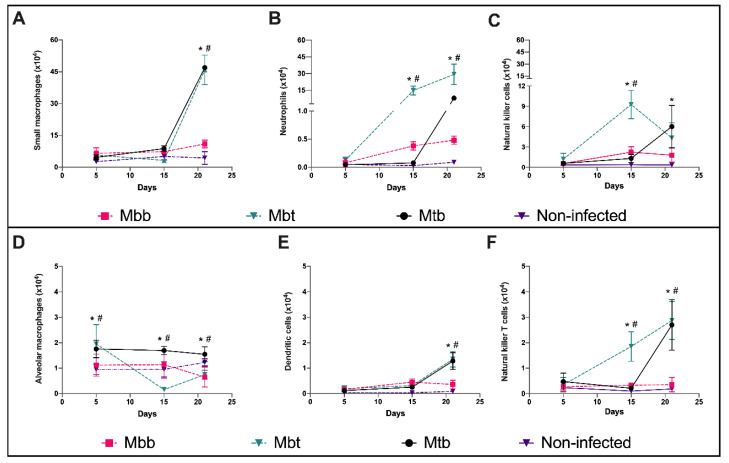
Kinetics of alveolar macrophages, monocytes, dendritic cells, neutrophils, and NK cells in the lungs of infected C57BL/6 mice. Lungs of mice not infected (non-infected) or infected with Mtb, Mbb, or Mbt euthanized at 5, 15, and 21 days p.i. were analyzed by flow cytometry. The results show the mean ± standard deviation (*n* = 6 per group) and significant differences (*p* < 0.05) between groups are shown as: * differences when compared with non-infected group; # differences between Mbt and Mbb groups. The kinetics of (**A**) total number of monocytes (small macrophages, F4/80^low^CD11b^low^CD11c^low^), (**B**) neutrophils (F4/80^+^CD11b^high^CD11c^−^Gr1^+^), (**C**) NK cells (CD3^−^NK1.1^+^), (**D**) alveolar macrophages (F4/80^+^CD11b^+^CD11c^−^), (**E**) dendritic cells (F4/80^+^CD11b^+^CD11c^+^), and (**F**) NKT (CD3^+^NK1.1^+^) are shown.

The total number of monocytes (small macrophages) recruited into the lungs of mice infected with Mtb and Mbt significantly enhanced 21 days p.i. when there was twice as much as seen in the lungs of mice infected with the isolate Mbb (Figure 5A). Mice infected with Mbt presented a significant but transient reduction in alveolar macrophages at 15 days p.i. (Figure 5D), while Mtb presented increased alveolar macrophage levels during all evaluated time points. The kinetics of dendritic cells (DC) in mice infected with Mbt or Mtb were similar. DC in lungs of infected mice from those two groups doubled by 21 days p.i. (Figure 5E).

The infection of mice with the Mbb isolate did not show an increase in neutrophils throughout the study period. However, in contrast, Mbt infection induced intense migration of neutrophils to the lungs of infected mice seen at 15 and 21 days p.i, while Mtb infection resulted in a significant neutrophil accumulation similar to Mbt infection, but this increase occurred only at 21 days p.i. (Figure 5B).

Mtb infection induced increased levels of NK cells by day 21 p.i while infection with Mbt induced higher numbers of NK cells at 15 days p.i. than Mtb infection did. In contrast, Mbb infection induced a discrete increase level of NK cells that remained constant through the studied period (Figure 5C). Mbt infection also induced increased levels of lung NKT cells at higher levels than Mtb-infected animals at day 15 p.i; however, later at 21 days p.i., both groups showed similar numbers of NKT cells in the lungs. Mbb infection did not alter the levels of NKT cells in the lung of infected mice (Figure 5F).

Overall, the innate immune response observed in mice infected with the Mbt isolate was similar to mice infected with *M. tuberculosis.* However, in Mbt-infected mice, neutrophils and NK cells migrated earlier to the lungs than mice infected with Mtb.

### 3.4. Modulation of the Acquired Immune Response

**Figure 6:**
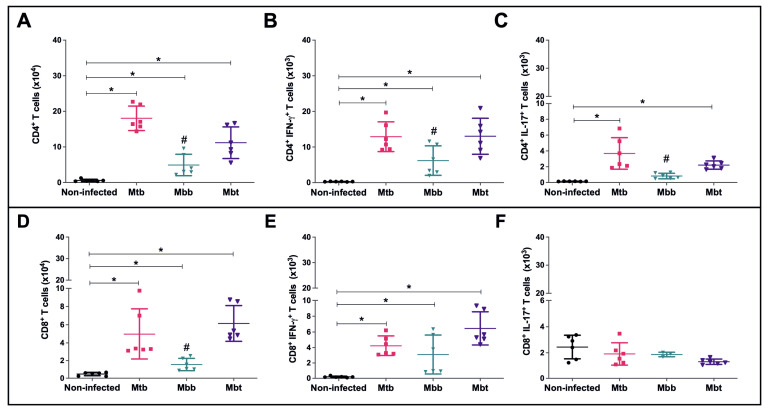
Subpopulations of T lymphocytes in the lungs of infected C57BL/6 mice. Lung cells from mice infected with Mtb, Mbb, or Mbt strains were euthanized at 21 days p.i. and analyzed by flow cytometry. The results show data from each mouse and the mean ± standard deviation per group (*n* = 6). (**A**) Total numbers of CD4^+^ T cells. (**B**) Total numbers of CD4^+^IFN-γ^+^ T cells. (**C**) Total numbers of CD4^+^IL17^+^ T cells. (**D**) Total numbers of CD8^+^ T cells. (**E**) Total numbers of CD8^+^IFN-γ^+^ T cells. (**F**) Total numbers of CD8^+^IL17^+^ T cells. Note that (**A**,**D**) are graphed as 10^4^ cells. Significant differences (*p* < 0.05) to the non-infected group (*) or between Mbt and Mbb groups (#) are shown.

Since Mbt infection induced differential levels of neutrophils and NK cells but resulted in early mice mortality, we questioned whether the animals infected with this isolate were able to produce an effective specific immune response. Twenty-one days post infection, CD4^+^ T lymphocytes were evaluated and compared among the different infected groups. As observed in Figure 6A, the infections were able to induce an increase in the numbers of CD4^+^ T cells in the lungs. However, the levels of CD4^+^ T cells induced by *M. bovis* isolate infection were lower than the ones obtained by Mtb infection (*p* = 0.04), while the CD4^+^ T cell levels induced by Mbt infection were higher than Mbb infection (*p* = 0.02). CD4^+^ IFN-γ T cells evaluated in an ex vivo assay showed that while the Mtb and Mbt infection induced higher levels of those cells, the animals infected with Mbb presented half of the number of cells seen in mice infected with Mtb or Mbt (Figure 6B). As described elsewhere [40], Mtb infection induced increased levels of Th17 cells (IL-17) as shown in Figure 6C. *M. bovis* infection induced a discrete enhancement of CD4^+^IL-17^+^ T cell numbers in the lung, but Mbt infection elicited almost four times the amount induced by Mbb infection (*p* = 0.022) (Figure 6C).

After 21 days of infection, all infected groups showed an increase in the population of CD8^+^ lymphocytes in the lung when compared with the non-infected group (Figure 6D). Interestingly, Mbt-infected mice was the group with the highest number of CD8+ T cells (6.15 ± 1.9 × 10^4^ cells). The infection with *Mycobacterium* isolates induced an increase in CD8^+^ lymphocytes positive for IFN-γ (Figure 6E). CD8^+^ lymphocytes positive for the cytokine IL-17 did not result in a significant change throughout the study period in infected mice from all infected groups (Figure 6F).

### 3.5. Kinetics of Pulmonary Pathology in Infection with M. bovis Isolates with Different PknG Proteins

**Figure 7:**
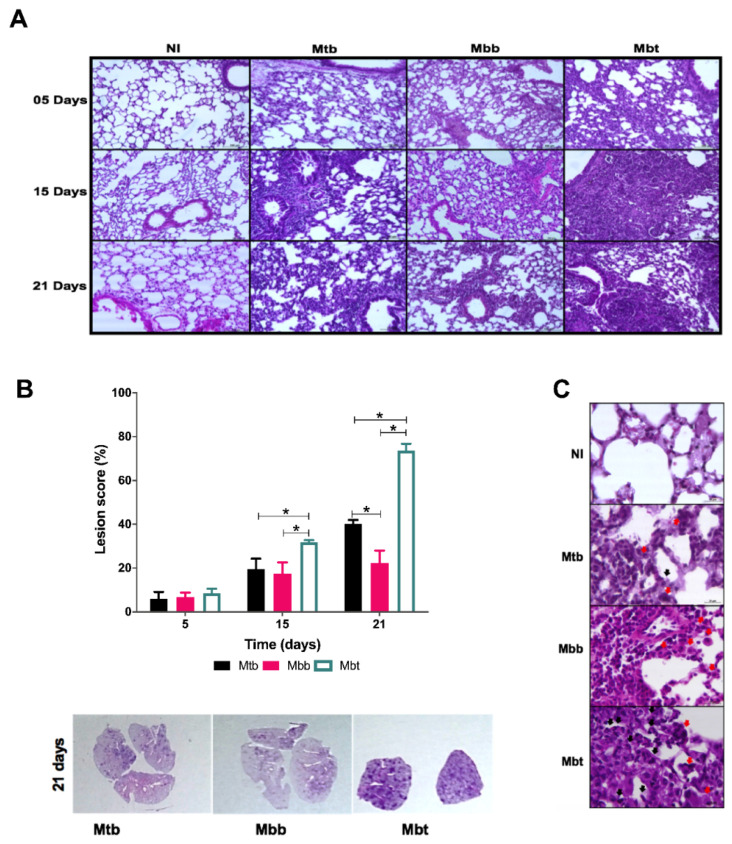
Lung histopathological analysis from C57BL/6 mice infected with Mtb and *M. bovis* isolates. C57BL/6 mice infected intravenously with 10^6^ CFU of the Mtb, Mbb, or Mbt strains were euthanized at 5, 15, and 21 days p.i. and their lungs were processed and stained with H&E for analysis. As the control, a healthy lung from a non-infected mouse injected with PBS (NI) was analyzed. (**A**) Lung sections from representative mice are shown (100× magnification). (**B**) Four to six random fields from each lung were evaluated to determine the percentage of the affected area of each mouse. The bars on the graph show the mean ± standard deviation of each group (*n* = 6); asterisk (*) denotes significant differences (*p* < 0.05) between groups. Representative lung sections at low magnification from each group 21 days post infection are shown. (**C**) Characteristic cells present in the lung lesions of mice at 21 days p.i. observed at 400× magnification, evidencing mononuclear (red arrow) and polymorphonuclear cells (black arrow).

Although no work has shown if PknG deletion in *M. bovis* or *M. tuberculosis* would affect the immune response, here we show that one single point mutation was associated with excessive innate immune responses. In order to evaluate if the increase in neutrophils seen among Mbt infected mice, as early as 15 days post infection, could have caused tissue damage that influenced the early mortality seen in that group, histological examination of the lungs was accessed. As anticipated, Mtb infection induced diffuse pneumonia, which intensified at 21 days p.i. (Figure 7A). Mice infected with Mbb isolate presented only peribronchiolar cell infiltrates with a predominance of mononuclear cells (15 days p.i.; Figure 7A). In contrast, the Mbt-infected mice showed intense diffuse inflammatory reactions throughout the lungs (Figure 7A). Histologic changes in the lungs of mice infected with Mbt could be seen in the very early stages of infection, with the presence of edema in the alveolar septum and diffuse cellular migration discreetly in some areas, which intensified along with the infection and culminated with the formation of structures similar to granuloma, some with central necrosis (Figure 7A). Parts of healthy lung tissue structures were lost due to disease progression and cellular accumulation (Figure 7A). The lungs of mice infected with Mbt for 21 days had a commitment of approximately 80% of their area with injuries in contrast with the lungs of mice infected with Mtb or Mbb that had a 40% or 35% damage area commitment, respectively (Figure 7B).

Although the infection with the Mbt isolate resulted in an immune response compatible with protection, mice presented lungs with an intensive accumulation of cells with segmented nuclei resembling neutrophils and mononuclear cells (Figure 7C, black and red arrows, respectively) that were associated with the necrotic areas. In contrast, the lungs of mice infected with Mtb or Mbb showed a predominance of mononuclear infiltrate (red arrows; Figure 7C) with very few polymorphonuclear cells near the lesions (black arrows; Figure 7C). Thus, the isolate Mbt harboring the PknG mutation presented higher virulence that was associated with an increase in neutrophils, pneumonia, and necrosis.

## 4. Discussion

Mutations, such as non-synonymous single polymorphisms (nsSNPs), accumulated during the evolution of the members of the MTBC can be responsible for the adaptations of these bacilli in their hosts [41]. The exchange between species of domestic or wild hosts also promotes the accumulation of nsSNPs [42]. The nsSNPs can affect metabolic pathways, such as the formation of the cell wall by causing a change in the host response to *M. bovis* infection [43]. Some proteins have important roles for infection, and thus may be more resistant to present nsSNPs. PknG was shown not to be essential for growth in vitro, nonetheless, it is important for mycobacterial virulence during infection [44]. Analysis of all PknG sequences from *M. bovis* strains showed only one reported mutation: an alteration of the amino acid arginine to proline, in the catalytic nucleus of this enzyme (at position 242; Figure 1). This mutation alters the structure of the protein by affecting the conformation of the ATP binding site cleft to be more open (Figure 2C), which could interfere with its function and consequently with the mycobacterial survival during macrophage infection. The deletion of PknG in mycobacteria (Mtb and *M. smegmatis*) rendered those bacteria susceptible to antimicrobial drugs, stress conditions such as acidic pH, hydrogen peroxide, starvation, or hypoxia [7,45]. Interestingly, the growth of the Mbt isolate under acidic conditions, with free radicals or antibiotics, showed that the Mbt isolate (R242P) presented a higher bacillary growth than the Mbb (Figure 3A–C). Overall, these results corroborate the observation that R242P mutation could induce a more open ATP binding site cleft, and this could result in increased kinase activity, conferring to the Mbt more resistance to stressful conditions. The enhanced growth of the Mbt isolate in stressful conditions could be a consequence of the physiological advantage of in vitro culture growth, except this was not the case (data not shown). Furthermore, the absence of the PknG did not affect the in vitro growth of *M. bovis* BCG or Mtb mutant strains in rich media [46], reinforcing the possible importance of PknG for mycobacteria growth under stressful conditions. The mutation in the PknG active site could result in differential expression of the enzyme during infection, but this was not the case once the expression of the *pknG* mRNA during macrophage infection was similar among the tested mycobacteria isolates (Figure 3D). These results indicate that the Mbt isolate has a higher survival capacity under adverse conditions, such as inside neutrophils and macrophages that create an environment rich in ROS and acid pH. For *M. tuberculosis*, it is well known that intracellular infection induces the recruitment of a V-ATPase proton pump to the phagosome to limit its acidification and to avoid bacterial death [5]. Recently it was shown that during macrophage infection, by exporting SapM and PknG in a SecA2 dependent manner, the phagosome maturation arrest by *M. tuberculosis* avoided the autophagosome formation and therefore mycobacterial death [5]. If PknG from *M. bovis* presents such a function it is not known, but the results presented here could indicate that these mechanisms could also be used by *M. bovis* Mbt. Conceivably, the R242P amino acid substitution in the PknG of Mbt isolate did not alter its expression level, as shown here. This could be related to improved secretion or activity (Figure 2) within the macrophage and to avoid phagolysosome fusion, but this must be further investigated.

The observed resistance of Mbt isolate to isoniazid could be due to the known mutations in the *katG*, *Inha*, *kasA,* or *ahpC* genes [47,48], but this was not the case as no resistance mutations were observed in those genes (data not shown).

Cowley et al. [49], in 2004, had already shown that PknG regulates glutamate/glutamine levels in response to in vivo *M. tuberculosis* infection using BALB/c mice, an important nutrient metabolism. Former work correlated PknG function to granuloma formation and resistance in a latent state of guinea pigs infected with *M. tuberculosis* [7]. Since we observed in mice infected with Mbt the presence of central necrosis (Figure 7A), reduction in alveolar macrophages (Figure 5D), increased neutrophil influx (Figure 5B), as well as increased extravasation of bacteria in the macrophage culture (Figure 3E), we presumed that this isolate had the capacity to induce more cell death. Necrotic cell death is a complex phenomenon involving several distinct mechanisms. Ferroptosis is a mechanism that has recently been associated with an increase in the severity of tuberculosis in several species [50]. Iron dependence for the induction of ferroptosis is highly relevant for Mtb infection, which is influenced by the availability of this bioactive metal [51]. The work of Amaral et al. [52] demonstrated that inhibition of the iron pathway by iron chelator induces inhibition of lipid peroxidation, suppresses necrosis in lung tissue, and leads to greater control of the Mtb infection. In fact, the addition of DFO in the culture of macrophages infected with the Mbt isolate demonstrated a reduction in cellular extravasation (Figure 3E, extracellular bacterial load), indicating a reduction in the number of macrophages killed during infection. Thus, ferroptosis appears to be one of the mechanisms in which the Mbt isolate induces macrophage death, in addition to providing the extracellular growth and spread of the bacilli and increasing ROS production by the Fenton reaction [53], and promotes tissue destruction after death by ferroptosis.

The mouse model of infection with different isolates allowed the evaluation of their pathogenicity. *M. bovis* strains with different virulence can present similar bacillary growth in the first months of infection in the murine model [16,40] and, apparently, *M. bovis* possesses enhanced capacity of growth in the mice when compared with Mtb [15]. Thereby, the higher bacillary load observed in animals infected with both isolates of *M. bovis* compared with Mtb cannot be associated with greater pathogenicity of the isolates but may be due to a better adaptation of the species to grow and survive in these animals. Despite the similar bacillary load observed in the infection by the two *M. bovis* isolates, the mortality of the mice infected with the Mbt isolate was significantly higher, indicating that the immune response induced by infection with this strain could be responsible for the animals’ early death.

Although populations of DCs and monocytes that have infiltrated in response to infection with the Mtb or Mbt strains were not significantly different during the infection, the alveolar macrophages and neutrophils showed different kinetics. The previous work by Andrade et al. [54] showed that isolates that have a higher virulence/pathogenicity induced higher macrophages death. This fact could explain the observed reduction in the alveolar macrophages in the lungs of mice infected with the Mbt isolate. We hypothesize that, in an attempt to return to homeostasis, the animals in this group increased the recruitment of circulating monocytes and neutrophils to the site. It is possible that the death of alveolar macrophages could also induce activation and presentation by DCs [55]. Previous work has also shown that necrotic lung damage by mycobacteria was associated with neutrophilia [56] and as seen in our study the emergence of necrotic lesions coincides with the greater influx of neutrophils to the lungs seen by flow cytometry analysis. It has been shown that neutrophils engulf bacilli, produce reactive oxygen species (ROS), and excrete neutrophils extracellular traps (NETs) [57] that were associated with necrosis. Both macrophage and neutrophils actively scavenging debris could have become infected [58], and probably in the lungs of Mbt-infected animals, the increased macrophage levels were not able to generate the adequate clearance of the lung tissue, maintaining the pneumonia and the granuloma central necrosis. Because the bacillary load was similar among the infected organs, we believe that the increased neutrophils and macrophage levels were associated with the lung lesions.

NKT cells exert a protective role in the innate immune responses against mycobacterial infection; they recognize *Mycobacterium-tuberculosis*-infected macrophages and produce interferon gamma to control the bacillary load [59]. NKT cells, as shown in the study of Chiba et al., become active, proliferate, and exert their effector function before the arrival of T cells during murine infection [60]. Thereby, NKT cells that migrated early to the lungs of Mbt-infected animals could be participating in the maintenance of a proinflammatory microenvironment and activating the macrophages and neutrophils in the lung by assisting with the aggravation of the damage in this tissue as seen in the histopathology. We have previously suggested that NK cells do not have a role in the direct control of the mycobacteria growth of Mtb [61], however, the contact between NKs and DCs during infection by *M. bovis* increases the production of IFN-γ, TNF-α, CCL2, CCL4, and CXCL8 [58]. The release of CXCL8 and CCL2 induce the recruitment of circulating neutrophils and monocytes [62]. Despite not having measured the levels of CXCL8 in this study, the kinetics of NK cells migration coincides with the increased migration of neutrophils to the lungs during Mbt infection.

Infection by the members of the MTBC induces increased migration of CD4^+^ and CD8^+^ T lymphocytes to the infected organs. The migration of these cells to the lung occurred in all infected groups, showing that the infection with Mbt, although presenting higher mortality, did not modulate the lymphocyte migration. Hypervirulent strains of *M. bovis* induced higher levels of proinflammatory cytokines, such as IFN-γ, IL-17, and IL-22, in mouse models as compared with less virulent strains [18]. Thus, the induction of pro-inflammatory cytokines may be a mechanism of virulence. Although we did not quantify the total cytokines, we observed an increase in the main cellular groups responsible for the production and secretion of these cytokines, such as Th1, T CD8^+^, and Th17 cells in all studied groups. Therefore, such cell populations alone were not responsible for the lung lesions that probably culminated with the death of mice. The increased production of IL-17 by CD4^+^ T cells might also contribute to the recruitment of neutrophils [63] that was initiated with the concomitant migration of NK and DC to the lungs.

It seems that the lifetime of infected animals was correlated with changes in lung tissue, such as necrosis and the appearance of granuloma-like structures. The histopathology of lungs from animals infected with Mbt showed granulomas, some with central necrosis, well structured, with extensive areas containing inflammatory infiltrate, which did not occur in the lungs of mice from the other groups. The ability to increase cell migration to the lung does not seem to be dependent on the bacterial load, but the virulence of *M. bovis* strain [17]. Thus, the different immune cell profiles that migrate to the interior of the alveoli, rather than the bacillary load, could be the cause of extensive diffuse pneumonia of mice infected with the Mbt strain that, in turn, was the main cause of mortality.

It is possible that other genomic differences could account for the observed virulence difference between the analyzed *M. bovis* strains. This was partially investigated by comparing the nsSNPs found in the *M. bovis* Mbt genome with the virulent and non-virulent strains. By this approach, four genes (*pknG*, *pks3*, *pks5*, and *mmpL4* genes) presented snSNPs exclusive of virulent strains (data not shown), while the position of *pknG* nsSNP was the only one at an important activity site. Other genetic differences might also contribute to the intense pathogenicity observed for *M. bovis* (R242P) that will need to be further studied by inserting the mutation in the wildtype strain.

The large percentage of damaged areas in the lungs of the Mbt group may have induced a severe respiratory deficiency that could be responsible for the mortality observed in those animals. Thus, the PknG R242P mutation could be related to an increase in protein activity rendering an acquired advantage for the *M. bovis* Mbt strain that strongly modulates the innate immune response increasing its virulence and pathogenicity.

## 5. Conclusions

PknG function is important for *M. bovis* pathogenicity and mutation in its catalytic domain increases mycobacteria virulence that culminates in severe pulmonary inflammation.

## Data Availability

The data are available, upon request, from the corresponding author.

## Supplementary Materials

The following supporting information can be downloaded at: https://www.mdpi.com/article/10.3390/microorganisms10040673/s1. Figure S1: Ramachandran plot of PknG proteins. Models of PknG proteins were evaluated before and after MD simulation. It was noticed that after MD initial steps, protein structure quality increased. Most of the outlier residues (outside delimited blue lines) are from the NORS domain (residues 1–75), described as disordered regions. Figure S2: RMSF and RMSD analysis through 200 ns of simulation. A. The root mean square fluctuation of protein from the Mbb strain has more match values in both replicas than protein from the Mbt strain. It seems that PknG from Mbt (replica 1) has higher residue fluctuations compared with the PknG Mbb replicas. On the other hand, the Mbt replica 2 has overall lower RMSF values than the PknG Mbb replicas. B. Root mean square deviation of both replicas from the PknG protein (Mbb and Mbt strains). The first frame of each trajectory was used as a reference for the RMSD calculation. On average, the results show no significant difference among the strains. Figure S3: Opening assessment of substrate binding cavity. To evaluate the opening of the substrate binding cavity, the distance between two residues was evaluated. A. Structure of PknG protein showing in detail residues chosen for analysis of distance. B. Minimum distance of Cys109-Asn346 through 200 ns of MD simulation of PknG from the Mbb strain. It is observed that only in the end of the simulation is there an opening of the substrate-binding cavity in replica 2. C. Unlike for the Mbb strain, it seems that for the Mbt strain the distance between Cys109-Asn346 is higher. It is noticed that there is no value lower than 1 nm after 75 ns of simulation, resulting in an overall more open conformation for substrate binding when compared with the Mbb strain.
