# Supplementary material for: Mycobacterium bovis PknG R242P Mutation Results in Structural Changes with Enhanced Virulence in the Mouse Model of Infection
Source: Microorganisms. 2022 Mar 22;10(4):673. doi: 10.3390/microorganisms10040673 (PMC9030157; doi:10.3390/microorganisms10040673)
Supplement: Supplementary file 1 [file microorganisms-10-00673-s001.zip › microorganisms-1628313-supplementary.pdf]

Supplementary Materials

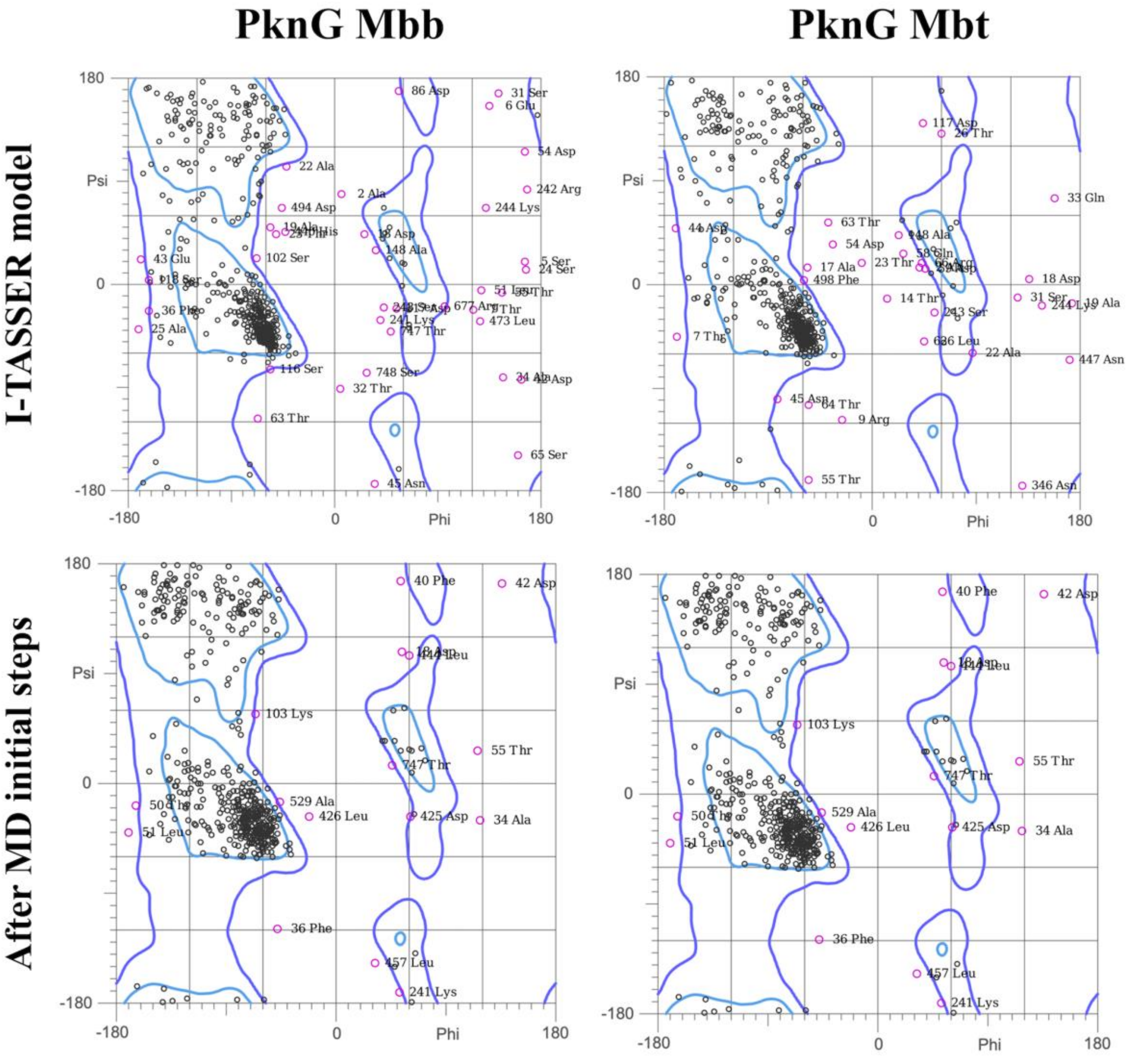

Supplemental Figure S1. Ramachandran plot of PknG proteins. Models of PknG proteins were evaluated before and after MD simulation. Light and dark blue lines represent the favored and allowed regions, respectively. After MD initial steps for PknG protein from the Mbb strain, the residues in favored (allowed) regions increased from 82.0 % (92.1 %) to 89.3 % (97.1 %). These values for PknG protein of Mbt strain were increased from 82.8 % (92.4 %) to 90.2 % (97.7 %) after initial MD steps.

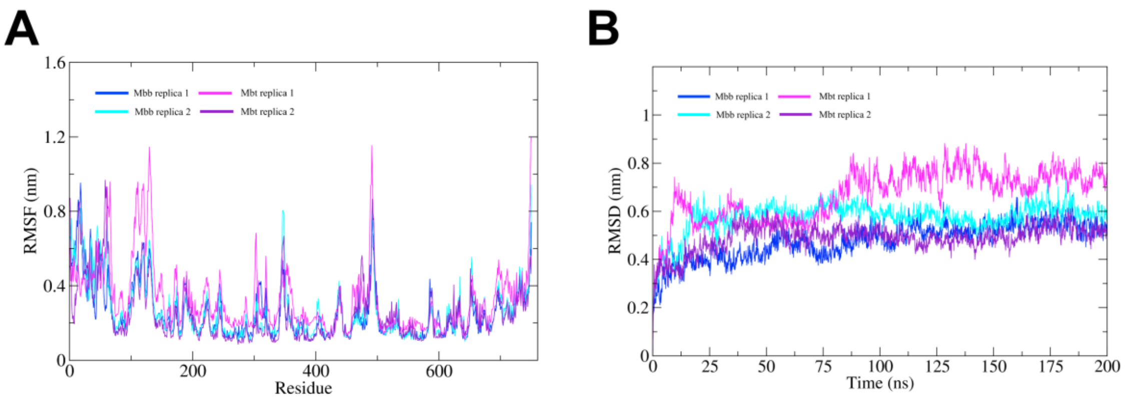

Supplemental Figure S2. RMSF and RMSD analysis through 200 ns of simulation. **A.** The Root Mean Square Fluctuation of protein from Mbb isolate has more match values in both replicas that protein from Mbt isolate. It seems that PknG from Mbt (replica 1) has higher residue fluctuations compared to PknG Mbb replicas. On the other hand, Mbt replica 2 has overall lower RMSF values than PknG Mbb replicas. **B.** Root Mean Square Deviation of both replicas from PknG protein (Mbb and Mbt isolates). The first frame of each trajectory was used as reference for RMSD calculation. On average, the results show no significant difference among isolate .

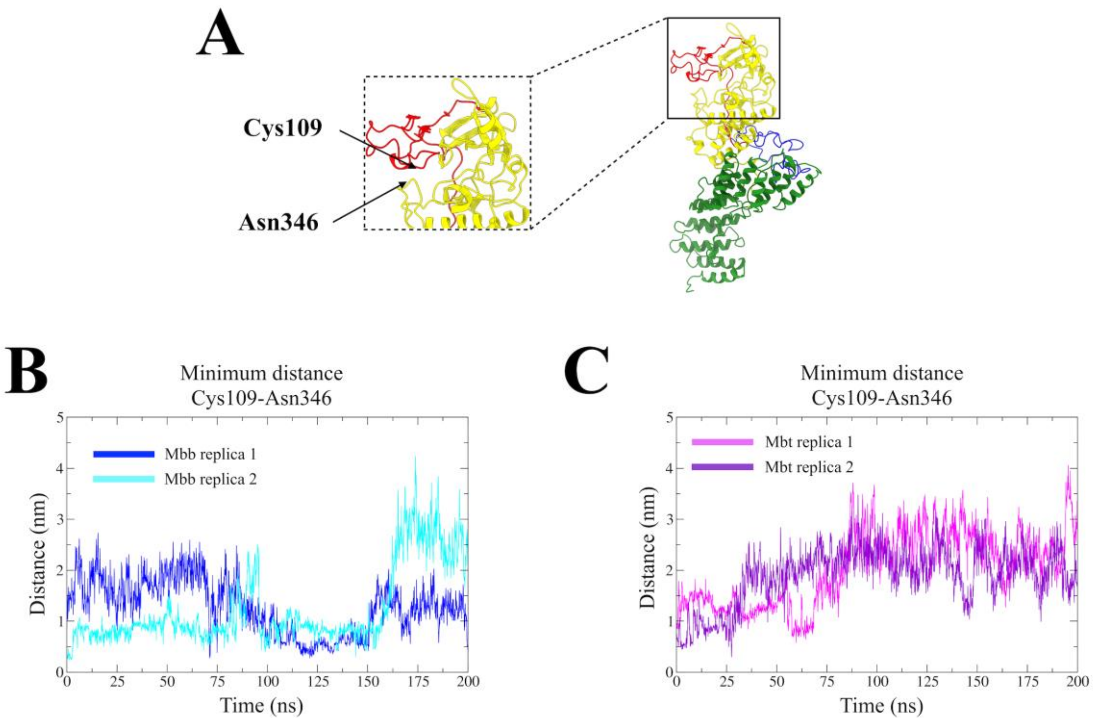

Supplemental Figure S3. Opening assessment of substrate binding cavity. To evaluate opening of substrate binding cavity, distance between two residues was evaluate. **A.** Structure o PknG protein showing in detail residues chosen for analysis of distance. **B.** Minimum distance of Cys109-Asn346 through 200 ns of MD simulation of PknG from Mbb isolate. It is observed that only in the end of simulation, there is an opening of the substrate binding cavity in replica 2. **C.** Unlike for Mbb isolate, it seems that for Mbt isolate distance between Cys109-Asn346 is higher. It is noticed that there is no value lower than 1 nm after 75 ns of simulation, resulting in overall more open conformation for substrate binding when compared to Mbb isolate.
